# First person – Alice Moncheaux

**DOI:** 10.1242/dmm.053224

**Published:** 2026-10-26

## Abstract

First Person is a series of interviews with the first authors of a selection of papers published in Disease Models & Mechanisms, helping researchers promote themselves alongside their papers. Alice Moncheaux is first author on ‘Increasing BIN1 or decreasing DNM2 worsens pathology in a mouse model of dysferlinopathy’, published in DMM. Alice is a postdoctoral researcher in the lab of Jocelyn Laporte at Institute of Genetics and Molecular and Cellular Biology, Illkirch, France, contributing to projects with strong clinical relevance, whether they focus on rare genetic diseases or on more common yet highly diverse conditions, such as cancer.

**Figure:**
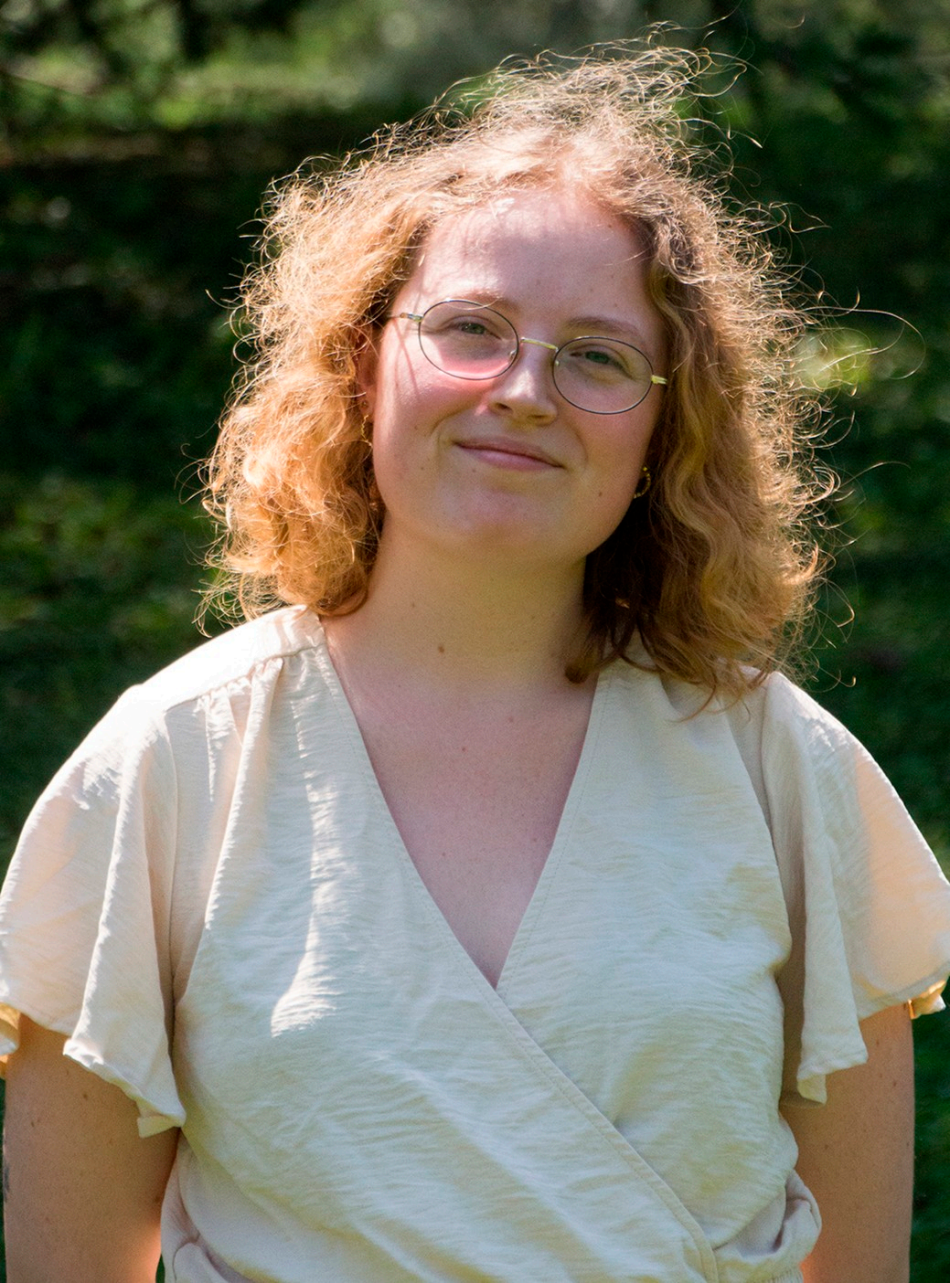
Alice Moncheaux

**Who or what inspired you to become a scientist?**

I think my first motivation was simply to satisfy my curiosity. I see biology as an infinite, largely uncharted territory, where every new discovery can help us understand the mysteries of life or lead to new tools and solutions for tackling diseases. In many ways, becoming a scientist feels like becoming an adventurer: there is always something new to explore, and you never quite know what you will discover next.

**What is the main question or challenge in disease biology you are addressing in this paper? How did you go about investigating your question or challenge?**

Dysferlinopathies are rare, progressive genetic muscle disorders that can cause fully ambulant individuals to become wheelchair bound within a decade. To date, no therapy is available on the market for patients with *DYSF* mutations. Since developing treatments for each rare disease is often less attractive to pharmaceutical companies, we investigated whether existing gene therapies could be repurposed by drawing on similarities between different muscle diseases, with the hope of identifying treatments that could benefit patients with distinct disorders.

To address this question, we used genetic crosses to modulate the expression of two genes in a mouse model recapitulating the histological hallmarks of dysferlinopathies. Although this work did not establish a new proof of concept for the treatment of dysferlinopathies, it uncovered a previously undescribed role for the protein dynamin 2 (DNM2) in the disease.

**How would you explain the main findings of your paper to non-scientific family and friends?**

It is widely recognized that some scientific discoveries arise from unexpected results. This work could be one such example: we aimed to treat a life-changing muscle disease using two treatments that were effective for other muscle diseases but surprisingly made the disease worse. In the end, this result allowed us to uncover a new piece of the puzzle of how muscle cells cope with damage.

… we aimed to treat a life-changing muscle disease using two treatments that were effective for other muscle diseases but surprisingly made the disease worse

**What are the potential implications of these results for disease biology and the possible impact on patients?**

Understanding the whole process responsible for membrane repair is one of the crucial axes to develop therapies targeting dystrophies. Our results participate in this scientific community effort.

**Figure:**
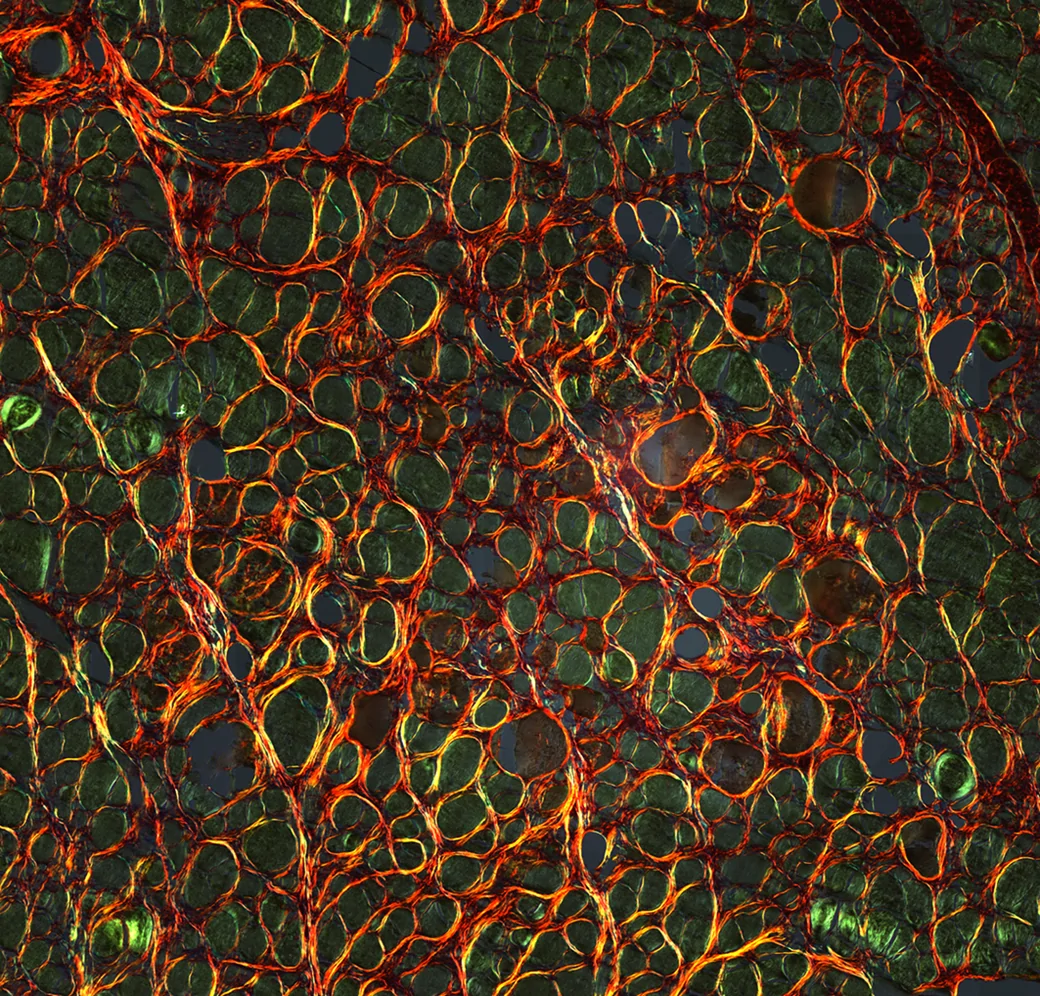
Muscle fibres trapped in expanded collagen matrix (Sirius Red staining under polarized light).

**Why did you choose DMM for your paper?**

Apart from the direct fit of our paper with the journal's focus on disease mechanisms, the Open Access was a great added value, as science advances through sharing.

**Given your current role, what challenges do you face and what changes could improve the professional lives of other scientists in this role?**

Sometimes in research, technical challenges and the need to dive deeper and deeper into the smallest details can make us lose sight of the bigger picture. For me, an important part of staying motivated as a scientist is meeting patients and hearing their stories. These encounters remind us of the people behind our research and give meaning to what we do. I believe that creating more opportunities for scientists to interact with patients and better understand their experiences could help us keep this sense of purpose in our daily work.

Sometimes in research, technical challenges and the need to dive deeper and deeper into the smallest details can make us lose sight of the bigger picture

**What's next for you?**

I will continue to work in translational medicine, while shifting temporarily my focus from investigating new therapies to optimizing treatment delivery, another important facet of the field.

**Tell us something interesting about yourself that wouldn't be on your CV**

When I am not exploring scientific leads, I go on hikes or ttRPG adventures. The quest never ends!
